# Bio-integrated carbon capture and utilization: at the interface between capture chemistry and archaeal CO_2_ reduction

**DOI:** 10.1038/s41467-024-51700-3

**Published:** 2024-08-29

**Authors:** Mads Ujarak Sieborg, Amalie Kirstine Hessellund Nielsen, Lars Ditlev Mørck Ottosen, Kim Daasbjerg, Michael Vedel Wegener Kofoed

**Affiliations:** 1 Department of Biological and Chemical Engineering, Aarhus University, Aarhus C, Denmark; 2 The Novo Nordisk Foundation CO₂ Research Center (CORC), Aarhus University, Aarhus C, Denmark; 3 Department of Chemistry, Aarhus University, Aarhus C, Denmark; 4 Interdisciplinary Nanoscience Center (iNANO), Aarhus C, Denmark

**Keywords:** Industrial microbiology, Applied microbiology, Metabolic engineering, Catalyst synthesis

## Abstract

Carbon capture and utilization (CCU) covers an array of technologies for valorizing carbon dioxide (CO_2_). To date, most mature CCU technology conducted with capture agents operates against the CO_2_ gradient to desorb CO_2_ from capture agents, exhibiting high energy penalties and thermal degradation due to the requirement for thermal swings. This Perspective presents a concept of Bio-Integrated Carbon Capture and Utilization (BICCU), which utilizes methanogens for integrated release and conversion of CO_2_ captured with capture agents. BICCU hereby substitutes the energy-intensive desorption with microbial conversion of captured CO_2_ by the methanogenic CO_2_-reduction pathway, utilizing green hydrogen to generate non-fossil methane.

## Abstract (web summary)

Existing carbon capture and utilization technologies are hindered by significant energy penalties. Here, the authors discuss the Bio-Integrated Carbon Capture and Utilization (BICCU) technology, which mitigates the energy penalties while generating valuable C1 and C2 products.

## Introduction

The global emissions of anthropogenic greenhouse gases have risen immensely during the past decades, with energy consumption from fossil fuel combustion being the main driver for around two-thirds of the total greenhouse gas emissions^1^. In 2022, the annual global greenhouse gas emissions from energy-related fossil fuel combustion and industrial processes reached 41.3 Gt_CO2-eq_, where carbon dioxide (CO_2_) emissions accounted for 89% of the total emissions (36.8 Gt_CO2_)^2^. However, the United Nations Climate Change Conference (COP28) declared the ‘beginning of the end’ of the fossil fuel era by the end of 2023^3^. The adoption of a renewable-based energy infrastructure has accelerated in the last decade with renewable electricity generation constituting 7858 TWh in 2021^4^. The transformation of the energy system towards heavy electrification has been envisioned as a significant factor in achieving carbon neutrality by 2050^5^. However, the hard-to-abate sector, including heavy transport and industrial processes, relies heavily on chemical energy carriers with a high volumetric energy density and remains difficult to electrify and decarbonize^6^. Here, Power to X (PtX) provides a potential solution that enables the conversion of electricity to chemical energy carriers, and combining it with Carbon Capture and Utilization (CCU) represents a critical technology to supply carbon-based energy carriers (e-fuels) for reaching the 2050 net zero emission target.

Various post-combustion carbon capture technologies are currently available, with chemical scrubbing being the most mature technology relying on solvent-based absorption and desorption conducted on the scale of megaton CO_2_ captured annually^7,8^. Conventional CCU with amine scrubbing consists of multiple steps, including CO_2_ capture by solvent absorption, CO_2_ release by heat desorption, CO_2_ dehydration and compression for storage and transport, and long-term storage or utilization in various downstream chemical and biological processes. Despite the many point sources of flue gas and a high potential for capturing CO_2_, conventional carbon capture with chemical scrubbing is attenuated by high energy penalties due to the dilute CO_2_ concentrations in flue gas, constituting up to 30% of the typical power plant output^9^. In conventional CO_2_ desorption from capture agents, the energy equivalent to the heat of absorption is added in a reboiler unit at the desorption column to induce the desorption and release of CO_2_. The typical thermal reboiler duty for the CO_2_ desorption column is 3.5–4.0 GJ t^−1^_CO2_^10–12^. Substantial research is therefore directed toward reducing the reboiler duty by modifying the capture agents and using blends that comprise capture agents with additives. Activators enhance the absorption and desorption kinetics and chemicals minimize the oxidative and thermal degradative behaviors^13^. The reboiler duty can furthermore be reduced by optimizing the desorption process parameters, such as adjusting operating pressures in the desorption unit and regulating reboiler temperatures^14^. Hence, multiple pilot-scale studies have demonstrated routes for reducing the reboiler duty of the desorption unit to a range of 1.9–3.6 GJ t^−1^_CO2_^15–17^.

In the future fossil-free society based on a circular economy, the captured CO_2_ is considered a valuable commodity that must be utilized to produce chemicals and fuels, which currently are fossil-based. However, carbon utilization preceded by carbon capture is limited by additional expenses for storing and transporting CO_2_, which requires dehydration to avoid corrosion and compression (~0.4 GJ t^−1^_CO2_)^18^. Thus, a group of new concepts based on integrated carbon capture and utilization (ICCU) is currently being developed, where captured CO_2_ is directly synthesized into valuable compounds simultaneously with the desorption from the capture agents. Hereby, the energy expenses for CO_2_ purification by desorption, transportation, dehydration, and compression are eliminated, improving the competitiveness of the capture process. The current ICCU processes include thermocatalysis^19^, electrochemical catalysis^20^, photoelectrocatalysis^21^, and biofixation with microalgae and cyanobacteria^22^. Nevertheless, several drawbacks have challenged the ICCU technologies. Thermocatalytic ICCU processes entail high temperatures and pressure to convert the CO_2_. Although isothermal operation can mitigate the energy penalty from the desorption, substantial efforts are required to identify a potentially stable and efficient CO_2_ sorbent capable of synergistically matching the reaction with the catalyst^23^. Furthermore, electrochemical catalysis offers the prospects of being conducted at milder temperatures, but this approach necessitates the utilization of expensive catalysts due to the chemical inertness of CO_2_^24^. Accordingly, additional gas conditioning of the raw flue gas will be essential to avoid poisoning the catalysts.

In contrast to chemical catalysts, biocatalysts such as methanogens can handle various contaminants such as hydrogen sulfide (H_2_S) and sulfur dioxide (SO_2_)^25^, which otherwise would deactivate the chemical catalysts used in both ICCU and CCU processes^26^. Methanogens belong to the domain Archaea and can be divided into three different physiological categories based on their substrate: hydrogenotrophic methanogens, methylotrophic methanogens, and acetoclastic methanogens^27^. The former is a key component in biological methanation, a robust Power to X (PtX) technology, where CO_2_ is reduced to methane (CH_4_) using renewable H_2_ from water electrolysis. Species important for the biomethanation process include *Methanobacterium, Methanobrevibacter*, and *Methanoculleus*, which all have been reported to be enriched during biogas upgrading, whereas the relative abundance of acetoclastic methanogens (species within the *Methanosarcinaceae* family) is usually experienced to decrease during biomethanation^28,29^. A common denominator for these microorganisms is their anaerobic trait, which renders them inhibited by oxygen (O_2_). Biomethanation has currently exclusively been utilized with feedstocks of biogas CO_2_^25,30,31^ and syngas CO_2_^32,33^ and has not been suited for direct conversion of flue gas CO_2_ due to the flue gas composition containing O_2_, which is detrimental to the obligate anaerobic methanogens. Furthermore, the CO_2_ is diluted with N_2_, which creates a dilute CH_4_ stream of <20% without any potential downstream application. The development of a technology that enables the biological utilization of flue gas CO_2_ would thus increase the potential of CO_2_ feedstock substantially, as biogenic CO_2_ from biogas only constituted 0.024 Gt_CO2_ by 2020 in Europe^34^, which is a mere fraction (~1%) of the 2.5 Gt_CO2_ emitted in Europe^35^. Targeting CH_4_ creates a versatile energy vector that indirectly electrifies the hard-to-abate sector while utilizing the already established natural gas grid infrastructures in the TWh magnitude^6^. However, the CH_4_ must comply with the standards of the distribution and storage grids, which are defined by the specific pipeline networks in the range 70–98% CH_4_ in the EU^36^.

This Perspective unfolds a concept for ICCU with simultaneous desorption and conversion of CO_2_ to CH_4_ based entirely on a microbiological driving force that enables biomethanation to be applied to dilute flue gases. Using methanogens for ICCU will ultimately alleviate the energy penalties and thermal degradation of the capture agent related to conventional carbon capture while reducing the number of process units required for CCU. This CO_2_ transformation route of bio-integrated carbon capture and utilization (BICCU) is currently demonstrated at a low Technology Readiness Level (TRL), but the concept, challenges, and prospects are presented here.

## Bio-integrated carbon capture and utilization

**Fig. 1:**
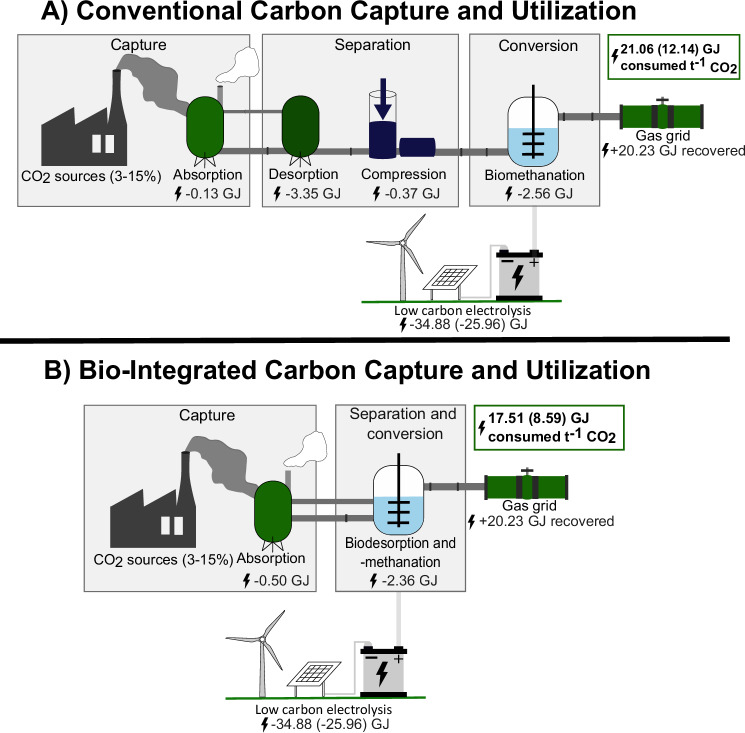
Process diagrams of conventional CCU and BICCU. **A** Conventional CCU. **B** BICCU. The conventional CCU process captures CO_2_ by absorbing it in capture agents and utilizes thermal energy for CO_2_ desorption. This process is followed by downstream compression and dehydration for storage and transport of the CO_2_ to a CO_2_ utilization unit of methanation for producing grid injectable CH_4_. In contrast, the BICCU process captures CO_2_ by absorbing the CO_2_ in a capture agent followed by combined microbial-mediated desorption and utilization by using renewable produced H_2_ to convert the CO_2_ directly into grid-injectable CH_4_. The energy balances for both systems are presented, with values expressed as GJ t^−1^ CO_2_ captured. Heat integration for the biomethanation process has not been considered. The numbers in parenthesis represent a scenario where electrolysis has no energy loss. All numbers are sourced from ref. ^38^.

The BICCU process captures the CO_2_ in an absorption column similar to the conventional CCU process but eliminates the demand for utilizing heat as a driving force for CO_2_ desorption. Instead, the proposed BICCU process relies on the biochemistry of anaerobic respiration of hydrogen- (H_2_) and CO_2_-consuming microorganisms—in this case, hydrogenotrophic methanogens. The microorganisms’ inherent enzymatic activity catalyzes the simultaneous desorption and conversion of CO_2_ into CH_4_ at ambient pressure and either mesophilic (20–45 °C) or thermophilic temperatures (45–60 °C). The result is a technology that makes CCU work with the CO_2_ gradient instead of against it by utilizing methanogens to maintain the gradient. The reducing equivalent for desorbing the CO_2_ from the capture agent and converting it into CH_4_ is H_2_ produced from water electrolysis with renewable electricity, thereby classifying it as a PtX technology. The biological desorption of CO_2_ regenerates the capture agents for recycling back to the absorption column for further carbon capture. Initial studies have benchmarked the performance and robustness of methanogenic microorganisms for the BICCU process by capturing raw flue gas from a biogas engine and converting it into CH_4_^37,38^. Several unit operations associated with CO_2_ desorption, conditioning, storage, and transport can be eliminated when applying simultaneous biological desorption and conversion. The resulting energy savings for the BICCU system with a capture unit, bioreactor unit, and electrolyzer unit will be ~3.6 GJ t^-1^_CO2_ corresponding to a 17–29% energy saving compared to the conventional CCU technology depending on whether the upstream energy balance for H_2_ production is included in the energy balance^38^ (Fig. 1).

The energy efficiency of the H_2_ production in water electrolysis (4.5–7.0 kWh m^−3^_H2_^39^) contributes substantially to the overall process energy loss (~52% assuming 4.75 kWh m^−3^_H2_) due to energy losses from the power supply heat losses in the rectifier, stack heat losses, energy consumption for auxiliaries for the balance of plant, and cooling requirements^38^. However, providing electrons in the form of H_2_ will be essential for reducing the captured CO_2_ regardless of the PtX technology applied. It is expected that the performance metrics of electrolysis will be improved by the rapid electrolysis maturation initiatives, such as the US Hydrogen Earthshot^40^, hereby supporting further development and maturation of PtX technologies like BICCU.

## Absorption and desorption

The CO_2_ point sources targeted for carbon capture, such as flue gas from burners and boilers, are diluted, since they are operated with atmospheric air containing excess oxygen to ensure clean burning, reduced soothing and CO emission. The resulting CO_2_ concentration is therefore in general 3–15% for combustion processes depending on the fuel type^41–43^. Other sources of CO_2_ relevant to the BICCU process could be industrial sources like non-combustion processes, such as cement plants, which emit 0.6 t CO_2_ t^–1^ cement produced^44^.

Due to the dilute nature of flue gasses, the CO_2_ must be separated from the exhaust or industrial gasses by capturing it in an absorption column containing a solvent that selectively interacts with the CO_2_ by chemical absorption. The CO_2_ absorption from flue gas could, in principle, be achieved with the intrinsic physical CO_2_ absorption with water, where weak molecular forces of Van der Waals or electrostatic interactions absorb the CO_2_. However, according to Henry’s law, the thermodynamic equilibrium between CO_2_ in the gas and aqueous phase will exhibit an absorption capacity that is linearly correlated with the partial pressure of CO_2_^45^, which would require a CO_2_ concentration of >59.3% CO_2_ to be cost-effective^46^. Instead, chemical absorption with a capture agent that selectively interacts with the CO_2_ can considerably enhance the CO_2_ absorption kinetics and capacity. These capture agents possess a strong affinity for reacting with CO_2_ to form chemical intermediate compounds that reversibly capture the CO_2_.

The most widely employed capture agents for chemical absorption are amines, which can react with CO_2_ in an acid-base reaction by two different reaction pathways. Non-sterically hindered primary and secondary amines react with CO_2_ in a two-step mechanism, where CO_2_ is initially converted into a zwitterion intermediate, which becomes deprotonated by an additional amine and thus proceeds to form a stable carbamate limiting the stochiometric CO_2_ loading to 0.5 mol_CO2_ mol_amine_^−1^ (Eq. 1). In contrast, the mechanism of CO_2_ capture by tertiary amines relies on the amines base-catalytic effect, which hydrates CO_2_ to produce bicarbonate (HCO_3_^−^) in a stochiometric CO_2_ loading of 1 mol_CO2_ mol_amine_^−1^ (Eq. 2).

1 $${{\rm{C}}}{{{\rm{O}}}}_{2}+{2{{\rm{R}}}}_{2}{{\rm{NH}}} \, \rightleftharpoons \, {{{\rm{R}}}}_{2}{{\rm{NCO}}}{{{\rm{O}}}}^{-}+{{{\rm{R}}}}_{2}{{\rm{N}}}{{{\rm{H}}}}_{2}^{+}$$

2 $${{{\rm{CO}}}}_{2}+{{{\rm{R}}}}_{3}{{\rm{N}}}+{{{\rm{H}}}}_{2}{{\rm{O}}} \, \rightleftharpoons \, {{\rm{HC}}}{{{\rm{O}}}}_{3}^{-}+{{{\rm{R}}}}_{3}{{\rm{N}}}{{{\rm{H}}}}^{+}$$

**Fig. 2:**
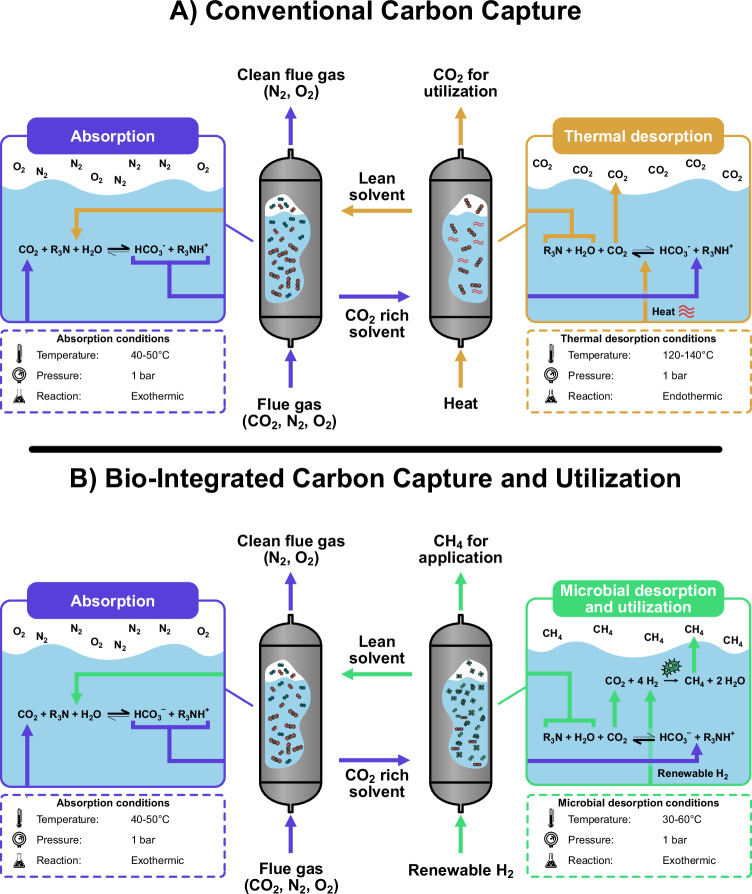
A schematic of the desorption process in conventional thermal-based carbon capture and the microbial-mediated BICCU process. **A** In the conventional carbon capture using a tertiary amine, the CO_2_ is absorbed in the absorption unit and transported to the desorption unit, where heat pushes the equilibrium toward CO_2_, thereby regenerating the amines. The liberated CO_2_ is sent for downstream compression, while the regenerated amines can be recirculated to the absorption column. **B** In the BICCU process, the CO_2_ is also captured by amines in the absorption unit and transported to the desorption unit, where microorganisms pull the equilibrium toward CO_2_ by continuously converting it to CH_4_ while regenerating the amines.

The CO_2_ absorption with amines is an exothermic process, and adding heat to the system reverses the reaction and pushes the equilibrium toward the release and desorption of CO_2_ by disrupting the chemical interactions between the capture agent and the CO_2_^47^ (Fig. 2A). For primary and secondary amines, the capture agents interact with the CO_2_ through covalent bonds, whereas tertiary amines elicit a shift in the chemical equilibrium to CO_2(aq)_/HCO_3_^−^_(aq)_^48^. The energy required for the release of aqueous or covalently bound CO_2_ is equal to the heat released by the exothermic CO_2_ absorption in the absorption unit. However, the heating requirement for the desorption of CO_2_ (reboiler duty) in conventional carbon capture is not limited to the heat of reaction for desorption, since the capture agent is in an aqueous solution that must be heated up to effectively reverse the CO_2_ binding. The heating requirement for stripping the CO_2_ from the aqueous solution can thus be designated to the sum of three principal components^49^ (*q*_reb_ in Eq. 3). The heating requirement for 1) the heat of reaction required to reverse the equilibrium and release the CO_2_ from the capture agents by disrupting the chemical interactions between the capture agent and CO_2_ (*q*_abs_), but also 2) the sensible heat, which corresponds to the heat required for increasing the temperature of the CO_2_ rich aqueous amine solution to the required regeneration temperature (*q*_sens_), and 3) the latent heat of vaporization of the water in the aqueous amine (*q*_vap_). Here, the heat of vaporization constitutes a significant energy sink in the conventional carbon capture and in the case of the primary amine, monoethanolamine (MEA) constitutes ~80% of the reboiler duty energy distribution^50^.

3 $${q}_{{{\rm{reb}}}}={q}_{{{\rm{sens}}}}+{q}_{{{\rm{vap}}}}+{q}_{{{\rm{abs}}}}$$

Thus, the capture agent’s high affinity toward CO_2_ consequently entails a high energy requirement to push the equilibrium toward liberating the CO_2_ to create a purified CO_2_ stream. Furthermore, energy must be invested for downstream storage and subsequent conversion of the purified CO_2_, which challenges the economics of CCU. In contrast, the BICCU process presents an alternative solution to eliminating the reboiler duty, where the CO_2_ in the carbonate equilibrium is utilized as a reactant for conversion into CH_4_ by using reducing equivalents of H_2_ to disrupt the chemical interactions between CO_2_ and the capture agent by the biomethanation reaction (Eq. 4).

4 $$4\,{{{\rm{H}}}}_{2}+{{\rm{C}}}{{{\rm{O}}}}_{2}\to {{\rm{C}}}{{{\rm{H}}}}_{4}+{2 \, {{\rm{H}}}}_{2}{{\rm{O}}}$$

The inherent metabolic activity of the hydrogenotrophic methanogens can convert the dissolved CO_2_ into CH_4_. Due to Le Chatelier’s principle, the bicarbonate equilibrium is shifted to the reactant side, hence desorbing more CO_2_ from the capture agent. Facilitating the CO_2_ desorption with a bio-mediated conversion mechanism enables the regeneration of the capture agents, which can be circulated back to the absorption column (Fig. 2B).

Transforming the conventional carbon capture with amines based on thermal desorption to a microbial-mediated desorption process in the BICCU process will reduce the energy requirements significantly since the desorption occurs at lower temperatures of <60 °C, where the sensible heat (*q*_*sens*_) and heat of vaporization (*q*_*vap*_) will become negligible. Low-temperature desorption has been researched for many years and can be achieved through utilizing e.g., biphasic thermomorphic amines^51^. However, although these low-temperature desorption processes for the liberation of CO_2_, including BICCU, reduce *q*_*vap*_ and *q*_*sens*_, energy is still needed for the endothermic desorption of CO_2_ (*q*_*abs*_). Therefore, BICCU has the additional advantage that the required energy for the endothermic desorption of CO_2_ can be supplied via the exothermic biomethanation process. The majority of the energy introduced to the bioreactor as H_2_ is conserved by the methanogens in the form of CH_4_ (~79%) whereas the remaining ~21% of the energy, corresponding to ∆*H*_298K_ = −165 kJ mol^−1^_CO2_^52^, is either converted to heat due to the exothermic nature of the methanogens or will become available for the production of biomolecules through assimilatory processes. Studies have shown that in contrast to the aerobic metabolism of C6 sugars, hydrogenotrophic methanogens are entropy-driven and mainly produce heat over biomolecules^53^—a phenomenon also observed in pilot-scale biomethanation reactors supplied with gaseous CO_2_ and H_2_^52^. The thermophilic conditions at ~50 °C required for the bioreactor thus couple remarkably well with the temperature conditions of 50 °C in the absorption unit due to the exothermic CO_2_ absorption. The advantage of combining capture chemistry and biology through BICCU is, therefore, not only to reduce the energy cost for CO_2_ liberation, but also to provide a potential energetic integration of chemical CO_2_ scrubbing with exothermic bioreactors.

The development of aqueous solutions of amines as an absorption medium for conventional carbon capture has been ongoing since the process development in 1930^54^, emphasizing various capture agent characteristics that render the capture process economically feasible and environmentally friendly. Substantial research efforts in reducing capital expenses have involved increasing the CO_2_ absorption kinetics and net-CO_2_ capacities of the capture agent, which reduces the size requirement of the absorption unit^55^. Simultaneously, efforts in reducing the operating expenses (OPEX) have been approached by reducing the enthalpy of the reaction and increasing the concentration of aqueous amines to minimize the energy lost for reversing the equilibrium to desorb CO_2_ and for heating and vaporizing water^56^, and solvent losses through oxidative and thermal degradation^57^. A knowledge spillover can thus be exploited for the maturation of the BICCU technology by utilizing the comprehensive research effort within conventional carbon capture with amine scrubbing. However, combining capture agents and microbial catalysts also requires novel developments within microbiology and capture chemistry.

In general, the carbamate formation involving covalent interactions between the primary/secondary amines and CO_2_ entails high absorption rate kinetics, but with correspondingly high enthalpies of reaction required for CO_2_ desorption. Tertiary amines are, in contrast, associated with lower enthalpies of reaction and higher CO_2_ absorption capacities but are limited by slow absorption kinetics^58^. Much attention has been given to addressing this trade-off between capture rate and energy demand through the development of novel capture agents and blends^59^, which will also dictate the efficiency of the BICCU process and become essential design criteria for capture agent selection, as listed in Table 1.

**Table 1:** Selection criteria for the CO_2_ capture agents in conventional solvent-based carbon capture and BICCU

General design parameters for CO_2_ capture agents	Conventional carbon capture (Heat-mediated desorption)	BICCU (Bio-mediated desorption)
- Fast CO_2_ absorption kinetics - High net-CO_2_ capacity - Low enthalpy of reaction	- High *T*_max_ for thermal degradation - Low amine volatility - Low aerosol formation - Low nitrosamine formation - Low latent heat of vaporization - Low sensible heat requirements	- Bioavailable CO_2_ binding - Low cellular toxicity - Biologically stable to avoid microbial decomposition

In conventional carbon capture, the high temperatures applied in the desorption unit necessitate critical considerations of the thermal stability (*T*_max_) of the capture agent. The thermal stability is directly related to the capture agent’s resistance to thermal degradation^60^, amine volatility, and aerosol formation^61^. The BICCU concept eliminates the desorption unit and the associated high temperatures and could potentially reduce the environmental and health concerns of conventional carbon capture and the OPEX associated with replacing the degraded amines. Another significant environmental concern with conventional carbon capture is the formation and accumulation of carcinogenic nitrosamines from accelerated thermal and oxidative amine degradation at high temperatures in the desorption unit, and amines reacting with NOx impurities from the flue gas in the absorption unit^62^. However, the high temperatures in the desorption unit have also been found to partly mitigate nitrosamine accumulation by further decomposing the nitrosamines thermally^63^. The multiple pathways to nitrosamine formation and degradation thus require further research to evaluate the fate of nitrosamines in BICCU.

When designing and developing capture agents for the BICCU process, thermal stability is no longer an essential design criterion (Table 1). Instead, the bio-mediated desorption requires capture agents that are non-inhibitory to the microorganisms, recalcitrant to microbial degradation, and employ weak CO_2_ bonding, such as bicarbonate formation with tertiary amines, to enable the bio-mediated CO_2_ stripping for capture agent regeneration. However, to design and develop suitable candidates of capture agents for the BICCU process, a further understanding of the microbial driving force for desorbing the CO_2_ is required.

## Microbial CO_2_ desorption mechanism from a tertiary amine

**Fig. 3:**
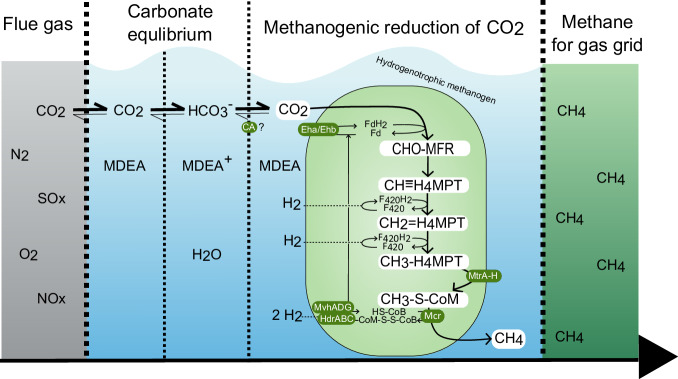
Proposed mechanism of methanogenic desorption and CO_2_ conversion. The mechanism is exemplified by MDEA as the capture agent and a hydrogenotrophic methanogen without cytochromes. The microbial conversion of the CO_2_ keeps the partial pressure low, which shifts the equilibria to the right and pulls the CO_2_ from MDEA. A simplified version of the CO_2_-reduction pathway shows the stepwise C1 reduction to CH_4_, with the C1-intermediates being highlighted by white boxes. The CH_4_ separates naturally into the gas phase and can be used for gas grid injection. MDEA is not metabolized by microorganisms and can thus be recycled to the absorption column.

To advance the development of the BICCU process, it is essential to understand the underlying mechanisms that drive CO_2_ desorption by hydrogenotrophic methanogens. The mechanism of simultaneous microbial desorption and conversion is thus theoretically explained and exemplified by applying the tertiary amine MDEA as a capture agent. During absorption, MDEA base-catalyzes the hydration of CO_2_, forming HCO_3_^−^, which is in equilibrium with small amounts of soluble CO_2_ (i.e., the carbonate equilibrium, Eq. 2). The hydrogenotrophic methanogens convert the soluble CO_2_ intracellularly by the CO_2_-reduction pathway, where they utilize inherent enzymes encoded by their genome to convert CO_2_ to CH_4_ with H_2_ as the electron source (Fig. 3).

The CO_2_-reduction pathway is well-described in literature^64,65^, but is here summarized due to its importance in the BICCU process. Once the captured CO_2_ enters the cellular cytoplasm, it undergoes a series of step-wise reductions by cytoplasmic electron carriers, which receive electrons from H_2_ supplied from water electrolysis using renewable power. The C1-intermediates are transported in the cell by the coenzymes, resulting in the formation of CH_4_ by the reduction of methyl-CoM by methyl-CoM-reductase. The resulting heterodisulfide compound (CoM-S-S-CoB) is recycled by a cytosolic hydrogenase-hetero-disulfide reductase complex (MvhADG-HdrABC). This exergonic reaction is thermodynamically coupled with the endergonic reduction of ferredoxin, which facilitates the first step in the CO_2_-reduction pathway, making the CO_2_-reduction pathway a cyclic process. An electrochemical gradient is maintained across the cell membrane throughout the process by enzymes such as MtrA-H and the Eha and Ehb complexes, resulting in ATP synthesis and energy conservation (Not shown in Fig. 3). The partial pressure of CO_2_ is thus kept low by the microbial CO_2_ consumption and shifts the carbonate equilibrium according to Le Chatelier’s principle, which enables the BICCU technology to work with the CO_2_ gradient instead of against it as in conventional amine-based carbon capture.

The shift in carbonate equilibrium in the BICCU process may be further mediated biologically by cellular carbonic anhydrases (CAs), which catalyze the interconversion of CO_2_ and HCO_3_^−^. Although their physiological role in methanogens is yet to be confirmed, CAs have been speculated to facilitate CO_2_ transfer across the cell membrane and concentrate the CO_2_ intracellularly to ensure sufficient levels for subsequent enzymatic conversion^66^. Additionally, cytoplasmic CA could concentrate the inorganic carbon in the form of HCO_3_^−^, which is a substrate for the CO_2_ fixation enzymes in the anabolic methanogenic metabolism^67^. The hydrogenotrophic methanogen *Methanococcus maripaludis* has been reported to secrete extracellular CA, thus making it likely that other hydrogenotrophic methanogens may share this trait^68^. The presence of extracellular CAs could potentially accelerate the rates of CO_2_ conversion in the BICCU process, as they together with MDEA control the bidirectional shift in the carbonate equilibrium. Extracellular CAs would be recycled along with the microorganisms to the absorption column, where they could aid in capturing CO_2_ from flue gas, if stable at the alkaline pH^69^. However, further research is needed to clarify the individual impacts of MDEA and CAs on the carbonate equilibrium and to further characterize the importance of methanogenic CAs in terms of CO_2_ acquisition.

The rate of methanogenic substrate conversion can be described with Michalis Menten kinetics, with research so far mostly focusing on determining the kinetic parameters for H_2_ uptake^70^. However, the CO_2_ uptake is more of interest for the BICCU process, as it influences the lower limit for microbial CO_2_ desorption and the maximum CO_2_ conversion rate. One study reported the microbe-specific Michaelis Menten constant (*K*_*m*_) for *Methanobacterium congolense* to be 2.5 mM for CO_2_ conversion^71^. This suggests that the maximum CO_2_ conversion rate would be achieved at 5 mM CO_2_ and above. However, it was confirmed from initial studies on BICCU with a mixed microbial culture that the CO_2_ conversion rates will decrease with MDEA concentrations from 30 mM and below, suggesting a variation across methanogenic strains to metabolize the captured CO_2_ and that the CO_2_ concentration is indeed a limiting factor^37,38^. The potential for BICCU will, therefore, potentially depend on both the microbial species and the capture agent employed, as the CO_2_-loading and the ability to convert it are two crucial elements for the process.

## Rate and stability of the biological process

The biological conversion of CO_2_ and H_2_ to methane (i.e., biomethanation) is a widely researched process that can be operated under various conditions and reactor configurations, utilizing the robustness of mixed anaerobic microbial cultures for conversion of CO_2_ and upgrading of biogas^30,31,72^. Implementations at industrial scale include the 2.5 MW ex-situ biomethanation plant by Hitachi Zosen Inova Schmack GmBh in Switzerland^73^ and the 1 MW facility by Electrochaea Gmbh in Denmark^74^. The latter demonstrated a CH_4_ production capacity of 800 L_CH4_ L_reactor_^−1^ d^−1^, emphasizing that biomethanation is both scalable and can achieve high conversion rates^74^. Additionally, the biomethanation concept relies on renewable, regenerative, and eco-friendly microorganisms as biocatalysts, which enables long-term continuous operation as demonstrated for up to 1200 days with highly concentrated CO_2_ sources such as biogas^72^. However, the application is currently limited to streams with a high CO_2_ concentration and without O_2_ unless the BICCU technology is applied, and continuous operation of bioreactors with diluted CO_2_ as the substrate has thus only been demonstrated for up to 80 consecutive days^37^. The same continuous BICCU reactor experienced an enrichment of the genus *Paracoccus* when exposed to an O_2_-rich capture agent, indicating a potential biological O_2_ scavenging mechanism by utilizing electrons from H_2_. The use of complex cultures could thus be a way to alleviate O_2_-stress and maintain the anaerobic conditions necessary for biomethanation.

The H_2_ gas–liquid mass transfer rate is often the limiting factor in biomethanation processes, as H_2_ is poorly soluble in aqueous solutions, with a Henry coefficient of 0.00078 mol kg^−1^ bar^−1^ at 25 °C^45^. Different gas-liquid contactor technologies have been developed to enhance the gas-liquid mass transfer of H_2_ to the active methanogenic microorganisms, including gas diffusers for the creation of small gas bubbles^75^, gas-phase bioreactors^31^, and membrane bioreactors^76^. Alternatively to exogenous H_2_ addition, future developments in bio-electrochemical systems, where H_2_ is produced in situ^77^, could present a platform for BICCU.

Besides ensuring sufficient amounts of dissolved H_2_, it is also crucial to minimize inhibition of the hydrogenotrophic methanogens by compounds detrimental to their metabolism. Such compounds include hydrogen sulfide and carbon monoxide in conventional biomethanation processes, where methanogenic communities have been reported to be adaptable to syngas^32,33,78^, and to be able to coexist with sulfur oxidizers^79^. In addition to these toxic compounds, inhibitors relevant to the BICCU process include SOx and NOx from the industrial flue gasses. NO_2_ has been reported to dissolve as NO_2_^−^ and NO_3_^−^ in a 0.1 M MDEA solution^80^, with especially NO_2_^−^ showcasing toxicity towards methanogens, with the extent depending on the methanogenic species^81^. However, synergistic relationships between microorganisms in complex cultures may alleviate the accumulation of these contaminants by e.g., simultaneous denitrification and methanogenesis, which have been reported in anaerobic biofilm reactors^82^. Accordingly, a lab-scale BICCU CSTR reactor managed to operate for 25 days on flue gas containing 141 ppm NOx with a complex mixed culture^37^. Long-term operation is, however, needed to investigate whether NOx accumulates to inhibitory levels or whether microbial synergies are relevant in this context. SO_2_ is captured by tertiary amines as sulfite, which inhibits methanogenesis due to the inactivation of methylcoenzyme M reductase^83,84^. However, the enzyme sulfite reductase and its homologs have also been reported across methanogens, making them able to tolerate small SO_3_^−2^ amounts^85^. The current BICCU studies were conducted on flue gas with SOx compounds but it remains unexplored whether the presence of SOx is a problem for long-term operation. Besides SOx and NOx, the toxicity of the capture agent is also of high importance, as initial studies reported microbial inhibition when using MDEA concentrations above 70 mM^38^. The exact inhibitory mechanism is still to be reported, but methanogens can be inhibited by structural analogs of coenzyme M, the compound involved in the final step in methane formation, as well as by medium or long-chain fatty acids through disruption of the cell membrane^86^. Minimizing microbial inhibition is critical since the BICCU process should operate with the highest possible concentration of capture agent as it increases the CO_2_-loading in the process and hence the rate and capacity. Increasing the capture agent concentration can only be accomplished within the range compatible with biological activity. In engineered systems using the continuous addition of a CO_2_-rich capture agent, a high CO_2_ load can be maintained by adjusting the capture agent volume and the recirculation rate. However, lowering the hydraulic retention time of the CO_2_-rich liquid in the combined biomethanation-desorption reactor through an increased recirculation rate will risk washing out the microbes. Research has demonstrated maximum doubling rates of ~1.6 h with an 80:20 mixture of H_2_:CO_2_ for *Methanothermobacter thermoautotrophicus*^87^, which is a potential biocatalyst candidate often cultivated for biomethanation^88^. This will limit the potential CO_2_ load to 16.8 L L^−1^ d^−1^ for a 50 mM MDEA solution, which previously had been used for BICCU^37^. However, the capture agent may introduce less favorable conditions that reduce the growth rate of the methanogens, and a potential solution to mitigate the washout risk would thus be to decouple the hydraulic retention time and the microbial biomass retention time, e.g., by retaining the microorganisms in a biofilm or biostructures on a solid support. This would furthermore prevent biomass O_2_ exposure in the absorption column and reduce O_2_-inhibition. Accordingly, ongoing state-of-the-art research and development within conventional biomethanation already point toward trickle bed reactors, a three-phase reactor bioreactor type with a fixed solid bed of carrier materials for biofilm immobilization, as a suitable reactor candidate^31^. Utilizing a bioreactor with a primary phase of gas, where the microbial biocatalysts are immobilized in a self-produced matrix of biofilm induces several advantages such as 1) reducing the liquid film layer that creates H_2_ gas-liquid mass transfer resistance, 2) detaching the microbial retention time from the hydraulic liquid retention time, and 3) enabling a controllable gas retention time due to plug flow approaching gas flow conditions without requiring high auxiliary power consumptions for agitation and gas sparging^89^. Naturally, these bioreactor types that allow the microbial biocatalyst to be retained in the bioreactor serve as a promising platform for BICCU.

Besides exposure to toxic compounds, methanogens can also be inhibited by an unfavorable pH, as most hydrogenotrophic methanogens grow optimally within the pH range 6.8–8.5^90^. Due to the alkaline properties of most amine-based capture agents, the solvent pH will be reduced when enriched with acidic CO_2_, whereas the microbial CO_2_ conversion will increase the pH. A complete microbial conversion of the captured CO_2_ is desirable for the efficiency of the process but might not be achievable due to the pH reaching inhibitory high levels prior to full depletion. On the other hand, a high pH is favorable for CO_2_ capture as most amines are deprotonated above pH 10–11 and are more efficient at capturing the acidic CO_2_ in this configuration. It is therefore a trade-off between obtaining process conditions that fit both the physiological optimums of the microbial community and the CO_2_ absorption efficiency in the absorption column. The fluctuating pH will furthermore constitute a selection pressure in a mixed methanogenic community, as observed in a continuous lab-scale BICCU reactor. The relative abundance of the genus *Methanobacterium* increased during reactor operation, indicating tolerance to higher pH levels imposed by the CO_2_-depleted MDEA, making this genus especially interesting for the BICCU process^37^.

## Outlook

The maturity of a technology can be assessed by its TRL, with 9 being the highest and describing a system proved in its operational environment and 1 being the lowest with only fundamental principles having been observed and reported^91^. To date, the BICCU technology has only been reported in lab-scale proof-of-concept experiments, to a TRL of 2. However, a strong point of the concept is that it combines elements of mature technologies—amine-based carbon capture, and biological methanation. Both have a TRL of 7–9—which can accelerate the maturation and enhance the technology scale-up potential. Accumulated knowledge from the last century can thus be a stepping stone for developing BICCU technology, which relies on capture agent properties such as fast absorption kinetics, high CO_2_ capacities, and low heat of reactions.

The application of BICCU can be expanded to other CO_2_-consuming microorganisms than methanogens. It has already been shown that CO_2_ assimilation for biomass production by microalgae growth can be promoted when supplied with a capture agent (i.e., polyethylene glycol 200)^92^. However, microalgae require light for their photosynthesis, resulting in complex photoreactors with a large footprint. Another naturally occurring bioprocess with CO_2_ as a reactant is homoacetogenesis, where homoacetogens such as *Clostridium* and *Acetobacterium* produce acetic acid from CO_2_ and H_2_ by the Wood-Ljungdahl pathway Acetic acid is a platform molecule that can be converted into single-cell protein (i.e., power-to-protein^93^) or used as a building block for acetyl-CoA-derived chemicals^94^. The global demand for acetic acid reached 14 million tons in 2019, with the majority being produced by methanol carbonylation^95^, making new sustainable production routes necessary. However, acetic acid is non-volatile and would be contaminated with the capture agent in the aqueous phase, unlike CH_4_, which naturally separates into the gas phase. Furthermore, the shift in equilibrium shown in Eq. 2 results in H_2_O formation, which would dilute the soluble product, rendering the downstream processing difficult when targeting liquid-based products. Acetic acid production by BICCU is therefore restricted and requires future optimization to avoid costly downstream separation of the capture agent and acetate. The microbial CO_2_ conversion might not be restricted to the natural autotrophic pathways, as an artificial CO_2_-fixation pathway was recently designed, constructed, and demonstrated in *E. coli*, yielding acetyl CoA as the final product^96^. A combination of artificial microbial pathways and BICCU would facilitate the production of a wide range of molecules to valorize the captured CO_2_ under mild conditions. However, whether this heavily engineered strain is competitive with the established biological process chains for CO_2_ conversion remains to be seen.

The BICCU technology concept holds the potential to become a viable and sustainable system that exploits the CCU and PtX approach envisioned for future energy systems. The combined bio-mediated desorption and conversion of the CO_2_ eliminates the desorption unit, downstream CO_2_ processing for storage and transport, and consecutive processing for utilization, resulting in a simplified process and energy savings in the range of 17–29%^38^. Furthermore, the reduction of the temperature from 120–140 °C in the thermal desorption to 30–60 °C in the bioreactor would potentially impede the thermal and oxidative degradation of the amine, thus improving the environmental impact of the process. However, regardless of the concept’s potential, the development and integration of this biomethanation-derived technology face a critical challenge when full capture and utilization of industrial CO_2_ flue gas streams are targeted. The efficiency of the amine scrubbing is limited to the CO_2_ loading capacity in the liquid capture agent, which is regulated by the capture agent concentration. Therefore, considerable biological and technical optimization is still necessary to achieve an industrially relevant technology. Critical elements of future research include (1) identifying absorbents with higher methanogenic biocompatibility at elevated capture agent concentrations and (2) identifying the right microorganisms capable of stripping the CO_2_ from the capture agents. Furthermore, developing a combined CCU and PtX technology concept relies on expanding the PtX infrastructure to supply H_2_ from renewable sources to ensure that the CO_2_ emissions for the overall process are kept at a minimum. As low-emission H_2_ production projects are increasing globally^97^, it seems plausible to rely on renewable H_2_ as a reducing equivalent for neutralizing and utilizing the CO_2_ emissions from flue gasses.

In conclusion, BICCU is a promising technology concept for negating energy penalties in CO_2_ capture, as the thermal desorption unit is replaced by a bioreactor that serves as a multifunctional reactor for the simultaneous release and conversion of the captured CO_2_ to CH_4_ by hydrogenotrophic methanogens. To further advance the technology, future research should be directed toward identifying biocompatible capture agents and robust microorganisms as many traditional capture agents have inhibitory effects on the microbiome.
